# Mobile homes in the land of illness: the hospitality and hostility of language in doctor-patient relations

**DOI:** 10.1186/s13010-023-00131-x

**Published:** 2023-04-12

**Authors:** Stephen R. Milford

**Affiliations:** 1 Extra-Ordinary Researcher Department of Theology, North-West University, Potchefstroom, South Africa; 2 Researcher at the Institute for Biomedical Ethics, Basel University, Basel, Switzerland

**Keywords:** Doctor-patient relationship, Health communication, Hostility in hospitals, Levinas, Derrida

## Abstract

Illness has a way of disorientating us, as if we are cast adrift in a foreign land. Like strangers in a dessert we seek oasis to recollect ourselves, find refuge and learn to build our own shelters. Using the philosophy of Levinas and Derrida, we can interpret health care providers (HCP), and the sites from which they act (e.g. hospitals), as *dwelling hosts* that offer hospitality to strangers in this foreign land. While often the dwellings are physical (e.g. hospitals), this is not always the case. Language represents a mobile home of refuge to the sick. Using language the HCP has built a shelter so as to dwell in the land of illness. However, while hospitality is an inviting concept, it also implies hostility. The door that opens may also be slammed shut. This article explores the paradox of the linguistic mobile home offered to patients. It highlights the power of language to construct a safe place in a strange land, but also explores the inherent violence. It ends with an exploration of the ways language can be used by HCP to assist patients to construct their own mobile shelters.

## Introduction

Medicine is, by its very nature, a moral enterprise and one’s underlying philosophical assumptions about the relationship between health care professionals (HCPs) and patients has wide ranging implications for the practice of medicine [1]. Is the HCP like the all-knowing father who tells his children what is right (paternalism); perhaps like the baker or mechanic offering a product or service to those who need it, and if they do not like what is offered they can go somewhere else down the road? Or perhaps the relationship is like a contract between two equal partners, both equally responsible for upholding its obligations. What we think about this relationship will impact how we engage with it and ultimately how we practice the art of medicine.

Floriani and Schramm have pointed out the great advantage of viewing HCPs and the sites from which they work (hospitals, care homes, surgeries etc.) through the lens of the philosophical concept of hospitality [2]. It encourages openness, humility, and provides a worthy vision to strive toward. Indeed, the very word *hospital* derives from the same roots as the word *hospitality* and it is from these same roots that words such as *hotel, host,* and *hospice* have come into use*.* Interestingly, the Latin root *hospes* (and its Greek equivalent *xenodochium*) can refer to both the host and the guest – since a host is also a type of guest in their own home [2, 3].

What is perhaps troubling is that these roots also account for the anti-thesis to hospitality: *hostility* [4]. That hospital is a positive phenomenon is intuitive, but that it includes – out of necessity – hostility goes against our natural inclinations. Yet, both these concepts derive from common roots and are intimately entwined; like two sides of the same coin. This project will consider the intimate link between hospitality and hostility that inhabits the health care context. As HCPs, in their sites of care, act as hosts and patients as guests, both hospitality and hostility is experienced. While Floriani and Schramm have briefly highlighted some challenges to the hospitality provided by HCP in terms of the use of technology, routinization, medicalization and an increasing distancing within doctor-patient relationships, they have not explored the implied contradiction in hospitality. Specifically that hospitality and hostility go hand in hand.

This article will concentrate on this paradox, with a particular focus on medical language as both a site for hospitality and hostility. Briefly sketching Levinas’ phenomenon of meeting the Other and Derrida’s unconditional hospitality, we will consider language as a tool of habitation and the primary site from which hospitality is offered. We will provide a unique interpretation of the context of the HCP-patient relationship and end by exploring the ways in which language can act as a non-physical mobile home that offers both hospitality and hostility.

### Unconditional hospitality and the absolute priority of the other

Levinas’ seminal work *Totality and Infinity* (1961), as well as Derrida’s lecture *Hospitality* (1996) provide us with a philosophically rich description of the relationship between ourselves and others. In Levinas we (referred to as the Self) exist among elements that are alien to us. The Self is driven by a need to provide for its own security and future stability. Being cast out into this alien world, it seeks protection and shelter, it seeks a home. Through a process of habitation, the Self collects and recollects itself, coming to know both itself and the world around it. The result is an inhabited Self, a Self that dwells in the world and is able to respond, act and react to its external context. The dwelling is the site of refuge for the Self, a place for the Self to retire and regather itself as needed. Consequently, habitation is the necessary condition for all human subjective action in the world [5].

The process of habitation involves mastery of the elements around the Self Wild in [6]. The ability to comprehend, categorise, organise, and manipulate the external elements for the purpose of the Self is absolutely vital. While there are many tools at the disposal of the Self, it is arguable that one tool in particular stands out: language. Through language the Self is able to name the elements around it. This naming is more than simply symbolic representation of foreign objects. Names distinguish elements, placing them in categories and arranging them into an organised system by which the Self can make sense of the world and its relationship to that world. Consequently language is fundamental to both the Self’s understanding of the world and its understanding of itself.

Having created a dwelling within the world by utilising tools such as language (among others), the Self must make a decision. It may isolate itself, cut itself off from the external world, retreating to its dwelling. This, however, is ethically problematic for Levinas who considers this action to be ‘the absolute truth, the radicalism, of separation’ [6] that creates a ‘pagan shrine’ [5] to the Self. Rather, Levinas understands that the Self has a desire for the transcendent, what Levinas refers to as the ‘metaphysical desire’ [6]. The Self does not long to return to its own land, but has a longing for a land not of its birth, a desire that cannot be satisfied and which keeps the Self reaching beyond its dwelling. Thus, the only ethical course of action for the recollected Self is to look outward.

Since the Self shares the world with other Selves (referred to as the Other), and since these Selves are also reaching beyond their own dwellings toward the infinite, it is only inevitable that their paths will meet. Like ships passing on the sea, or caravans in the desert, the Self is bound to encounter the Other.

To Levinas the Other is entirely foreign, a stranger to the Self. They are absolutely other and cannot be encompassed into the Self’s frame of reference: ‘he and I do not form a number. The collectively in which I say “you” or “we” is not a plural of the “I”. I, you – these are not individuals of a common concept’ [6]. Furthermore, according to Levinas, the Other resists categorisation of genus. Their alterity is not dependent on a quality that either distinguishes them or unites them to the Self. The Other is not a simple negation of the Self – a Self that stands in opposition to the Self. Rather, the Other remains ‘infinitely transcendent, infinitely foreign; his face in which his epiphany is produced and which appeals to me breaks with the world that can be common to us, whose virtualities are inscribed in our nature and developed by our existence’ [6]. The Other who presents himself before the Self is a stranger, one who is free and over whom the Self has no power.

When presented with the Other, the Self can respond in two ways. They can consider the Other as any other object, subsume them under one of the Self’s categories and give it a place in the Self’s systematised world. In this way the Self manipulates the other for the Self’s own purposes, denying the Other’s alterity. To Levinas this would be an inappropriate response. Recognising the absolute otherness of the Other places a demand on the Self to respect the Other fully and in so doing acknowledge the ‘absolute priority’ [2] of the Other.

To Derrida, the absolute priority of the Other places upon the Self the duty of offering ‘unconditional hospitality’ [4]. This is not, as Kant would argue, philanthropy – love for the human being. This is a duty the Self owes to the other, a right the Other has simply because they share a common space [4]. The Self is obliged to open up their dwelling, become a host, invite the Other in as guest and offer them unconditional hospitality. To both Derrida and Levinas this is the only ethically appropriate response that takes seriously the phenomenology of the Other.

While this is a general picture of human existence, it is particularly pertinent to the context of HCPs and patients.

### Doctors and patients as hosts and guests

Illness is more than simply a pathological challenge. It is an ‘ontological assault’ [7] that ‘puts the whole fabric of the sick person’s life-world at risk’ [8]. In sickness the body turns against the self to become a tyrant that makes demands that must be listened to. The sick body changes one’s subjective experience of reality even in the little things. To the healthy person the toilet is but a few steps away, to the sick person it is a marathon’s distance. The healthy person experiences a freedom to go and do as they please (within reason) but the sick person loses a freedom that is closely associated with our understanding of being human. We are forced to share important decisions about what happens to us with another. In thisway sickness wounds our very humanity [1, 7, 8].

It is as if the sick person is a stranger in a foreign land, cast out to fend for themselves in a hostile environment. Like a thirsty desert traveller, they long for an oasis, a place where they can find respite, have their needs met and recollect themselves. They must navigate this alien landscape, learning to gain some control over the unfamiliar elements that are afflicting them. In this foreign land they seek out natives that have gone before them and inhabit the region.

The doctor, the nurse, the care home assistant, are such natives. Through their training and experience, HCP have learnt to dwell in the land of illness and disease. Using tools they developed over many years, they have obtained some level of mastery over the elements of disease. This mastery does not imply that they are able to cure all illnesses, merely that they have made a shelter which provides some level of protection, and from which they may act. Using instruments such as language, they have identified; quantified; and organised elements of illness and disease, subsuming these into their own categories so as to employ them for their own purposes. They are the *dwelling natives* in this foreign land through which the sick stranger must travel. If the patient is to transverse this region, they will need the hospitality of these hosts.

Within this paradigm, medical sites such as hospitals, doctor’s consultancy rooms, and hospices, act as hotels. They are places of refuge and shelter. HCPs, through their profession, declare that they have special knowledge and skill, that they can heal and help, and that they will do so in the patients best interest [1, 7]. In this they take the role of hosts, opening up their sites and inviting the sojourners (patients) in. They do so in a spirit of welcome, seeking to tend to their guests’ needs, offering a place for the sojourners to find relief. To the sojourners they offer the resources and means to dwell in this foreign land, to recollect themselves, make sense of the strange elements for themselves and, having established their own dwelling (temporary or permanent), to engage in subjective thought and action within the land of sickness and disease.

While the sites of hospitality are often physical – hospitals, surgeries, care homes etc. – they need not be. According to Levinas, one of the primary ways of response given by the Self to the Other is in linguistic exchange. In language the Self presents itself and its world in words. It offers these words to the Other to be shared. To Levinas, language does not exteriorize a representation that is pre-exiting in the Self. Rather it attempts to put into common understanding a world that belongs to the Self. It is an ‘action without action,’ a first action by which the Self offers the world to the face of the Other and asks them to respond. This act of generosity is an act of hospitality. It is a way of offering ‘the world possessed’ [6] to the guest. In this sense, Levinas claims it is the ‘first ethical gesture’ [6].

For Treanor this is the ‘mobile home of language,’ [9] the non-physical dwelling site of the host from which unconditional hospitality may be offered. By speaking to the Other, the host helps the Other ‘implace’ [9] themselves in this foreign land. Guests are able to use words, stories, narratives, and myths to find their rightful place, and to write the place into their own story, making the guest feel at home [9].

For the HCP this is particularly pertinent. [7] The medical world is filled with unique language and jargon by which medical professionals are able to describe, quantify and organise the world of illness and disease. Naming illness; speaking of treatments; using particular grammar, are all tools at the disposal of the medical professional to allow them to dwell in this foreign land. Yet these are not tools readily available to patients who often experience an information deficit about the diseases that affect them. This hinders their ability to express their own moral agency [7]. Strangers to the land of illness and disease, they have yet to acquire such tools and consequently often struggle to build a dwelling for themselves. HCPs offer the possibility of acquiring these tools, but at the same time present risks and challenges.

### The hospitality and hostility of medical Jargon

To Levinas, the Other has absolute priority. Consequently, when the Other present themselves to the Self, the Self must respond in a particular way. Before the epiphany of the Other, the Self can cling to the ignorance of its own freedom. It can perceive of the external elemental world as a world of objects. Yet when presented by the Other, it discovers that this is not the case, and its uncontained freedom is potentially unethical. In the presence of the Other, the Self becomes ‘ashamed’ [5] of its isolation and is morally obligated to respond with unconditional hospitality to the Other.

Unconditional hospitality, however, is impossible. Pondering Kant’s substitution of the word *hospitality* with the German *Wirtbarkeit,* Derrida explores the paradox [4]. To Kant *Wirt* carries the implication of a host/patron, a master of the house. This formulisation ‘violently imposes a contradiction on the very concept of hospitality’ [4]. As master of the house, The Self offers hospitality on the condition that the guest respects the host’s mastery of the home. This, to Derrida, establishes the conditional laws of hospitality: that the guest owes a duty to the host. Consequently, while there are duties for hosts and rights for guests, there are also rights for hosts and duties for guests [9]. This places hostile limits on the gift of hospitality being offered. The guest is welcome, but they are not fully welcome. They are free to make themselves at home, but not absolutely free. The hospitality is constructed in such a way as to limit the freedom of the Other and place them under the authority and control of the host. Derrida highlights the figure of a door to illustrate the point:

To take up the figure of the door, for there to be hospitality, there must be a door. But if there is a door, there is no longer hospitality. There is no hospitable house. There is no house without doors and windows. But as soon as there are a door and windows, it means that someone has the key to them and consequently controls the conditions of hospitality [4]

Here Derrida highlights the paradox of hospitality. A door is necessary for hospitality; it indicates a dwelling or shelter that offers protection from the elements. In its opening, it invites a guest in, encouraging them to enter and dwell with the host for a period. However, the door also indicates that there is no hospitality. It is a controlled threshold that must be crossed. The host decides who can and cannot enter, and how they should and must behave within. Boersma sees an implied violence in this paradox [10]. Hospitality and hostility go together. The open door points both to the invitation of welcome as well as the cruelty of exclusion.

Turning back to HCPs and their sites of hospitality we can see that this paradox is affirmed. Health care sites of hospitality (such as hospitals), proport to be places of hospitality. Indeed, in the overwhelming majority of cases they offer life-saving care in very difficult circumstances. Patients are invited in and given incredible hospitality. However, the conditions of entry to these sites are often strictly controlled, at times even with armed guards. Elsewhere we have spoken about this at length (Milford & Lorenzini, The Hostile Hospital, submitted).^1^ What we have not addressed is the non-physical sites of hospitality that permeate these spaces.

While HCPs have physical sites from which hospitality is offered – hospitals, surgeries, care homes etc. – one of their primary dwellings is non-physical: language. From this mobile home, the HCP offers to the patient (the guest) hospitality. They offer them a place of respite, a chance to recollect themselves, and the opportunity to obtain the tools (words and grammar) needed to build a shelter in the land of sickness and disease. Yet their mobile home, like any home, has doors that are both open and threatening at the same time.

It is open in its invitation. That HCP have a linguistic mobile home is attractive to patients. As sojourners through the land of illness and disease, patients seek the most hospitable dwelling. Proficient HCPs who have built sturdy dwellings – those based on knowledge and skill, including linguistic skill – are the most inviting. Sojourners will knock on these doors and request to enter. If entrance is granted, they may find comfort in the protection offered by the dwelling: in the HCPs ability to speak about their illness; to categorise it; describe it; and exercise a measure of control over the patients’ illness. With luck, the host will help the patient learn to use the linguistic tools of the medical profession so as to construct their own shelter. This is a moral obligation on the part of HCPs as they are bound by their profession (declaration of assistance) to help patients express their own moral agency [7]. For the most part, the HCP is only too happy to open their language home up to the patient, to invite them in and to assist them to be *implaced* for the duration of their stay in this foreign land.

HCP are regularly providing this type of hospitality and many of us have first-hand experience of this. Interestingly, the rise of modern media has offered a new and exciting way of extending this experience. For example, the hugely popular smartphone app *TikTok* has a number of popular HCP’s sharing their knowledge and training about illness and disease. Naturally this raises some ethical questions about the phenomenon of popularist medical content as a form of entertainment. Notwithstanding this, there are good examples of responsible doctors who provide empirically based content to the public in informative and entertaining ways. This includes not only facts and figures, but also the terminology of illness and the skills necessary to navigate the research. Two such examples come to mind: @dr_idz who has over 1.5 million followers and shares nutritional information by citing academic meta-studies, and @dr.karanr with 5 million follows who discusses general medical conditions using medical terms. These, and many others, make their language sites available to the public (including patients) who can easily access their expertise and learn how medical language is used to make sense of, and master, illness.

Nevertheless, that such HCP have a linguistic mobile home is in many ways a threat to the absolute priority and freedom of the Other. Not only can HCP withdraw this hospitality at any time – thereby placing patients in very vulnerable positions – but they can turn their tools and their skills into weapons of authoritarianism. Using medical jargon; grammar; and language, it is possible for HCP to treat patients as objects to be subsumed into the HCPs systems and used for their own purposes. Recent decades have seen a resistance to such paternalism [11–13] and while it is beyond the scope of this project to engage in extensive discussions based on empirical research on the practical consequences of our model here, a very brief discussion has some merit.

### The not so practical practicalities

The model of considering language as a mobile home in the land of illness should lead HCP to reflect on their practice of medicine in helpful ways. One naturally draws to mind the topic of medical jargon which has long been a well-known challenge in patient care [14–16]. Over the years there has been much research to demonstrate that its inappropriate use can hinder doctor-patient communication, [17] be perceived by patients as unprofessional, [18], and in some cases even cause significant anxiety to patients [19]. Yet research has shown that HCPs use terminology not understood by patients more than 80% of the time [20] and as much as seventy times per encounter [20, 21]. In up to 80% of the cases, HCP do not either explain these words nor repeat them [22]. Even in the midst of mounting evidence demonstrating poor performance by medical professionals in this area, HCPs continue to overestimate their ability to communicate effectively [23].

What is interesting is that sometimes HCP underestimate patient’s understanding of medical jargon [24]. In these cases HCP incorrectly assume that patients are ignorant of medical terms. The consequence can be a paternalistic relationship between patient and HCP as the HCP attempts to explain complex medical conditions using simplistic language that fails to convey crucial medical information. Importantly, such basic communication hinders patients’ ability to make sense of their condition, to express fully their experience, and to exercise a greater level of mastery over their afflictions. Without the linguistic tools, patients find themselves at the mercy of the hospitality of the HCP, unable to construct their own dwellings and exercise a measure of autonomy – a fundamental principle in biomedical ethics [25].

Providing these linguistic tools goes beyond simply using less jargon by attempting to colloquialise health care as some may suggest [1, 14]. Of course providing patients with jargon busting glossaries of terms used within a specific medical context is hugely beneficial—such as in the case of asthma [22, 26]. Yet one cannot help but wonder if more is needed. Over the last few decades much work has been done to address the challenges associated with HCP-patient communication. This includes a focus on cultural competencies [27] as well as training programmes that focuses on humility and cultural linguistics [28, 29]. In addition consultancy services such as Vitaltalk.org have focused on using evidence-based methods to equip clinicians to navigate difficult conversations. This is all welcomed.

Yet providing glossaries and training programmes – as very useful as they are – may not be striking at the heart of the problem. Often in our attempts to solve deep rooted challenges we seek out easily quantifiable solutions. This is especially true of the medical context with its emphasis on empirical research and positivistic methodologies. While such methodologies are entirely appropriate in the context of the efficacy of a treatment option, they are not appropriate to human relationships which are dynamic and deeply personal. Pellegrino and Thomasma have highlighted this challenge many years ago as they called for a philosophy of medicine that allows ‘nonmeasurable clinical factors and values [to] be treated with the same attention as clinical indicators of disease’ [7].

Since each act of medicine is aimed at restoring wholeness – understood differently for each particular patient [1, 7] – so too is each HCP-patient interaction unique. Training programmes can help HCPs by providing some of the theoretical framework necessary to improve HCP-patient communication, as well as some practical examples of how this is done. However, these may only go skin-deep. What may be helpful is for HCP to adopt a deep-rooted philosophy about the nature of illness and the role of the HCP. This will have practical implications that go beyond quantifiable training programmes.

For example, what does ‘consent’ really mean in the context of HCP-patient relationships? Pellegrino and Thomasma urges us to go beyond merely legal consent that satisfies the contemporary need for a paper-trail to show that a patient understood intellectually the words being used. In Pellegrino’s philosophy of medicine, consent is a moral obligation for the HCP to understand what ‘wholeness’ means for a particular patient [1, 7, 30]. This requires a deep understanding of the patient’s life-world as well as how this life-world inter-penetrates the life-world of the HCP. To achieve true consent a ‘clinical truth’ [7] must be reached whereby the HCP and the patient each have understood the other, the nature of the illness, the options available, what wholeness means for this patient, and consequently the most appropriate course of action. This cannot be achieved where language is a major barrier.

To achieve this clinical truth, HCP need to open up their linguistic homes, draw patients in, provide them with the tools to express their own life-world within this new context of illness, and assist them to become implaced through the use of medical language. This goes beyond de-jargonising medical language. In fact, it may require the opposite as medical language is a powerful tool used by HCPs to exercise some control over illness. Rather it requires HCPs to share their language, teaching patients how to use medical jargon (wherever necessary), all while being conscious of the implied hostility and inequality that is a consequential part of the HCP-patient relationship.

How exactly HCPs can teach patients to use medical language is dependent on the innumerable individual circumstances HCP face. Above we have noted some examples – providing a glossary, using social media to teach medical linguistics, or enlisting the services of training consultants. Ultimately however, one must go deeper. As we reflect on our very nature – especially as these come into contact with the realities of other Selves – we reflect on our practices in rich ways. Reflecting on patients as guests and HCPs as hosts of mobile homes in the land of illness may stir within us a deep philosophy that drives individual practices aimed at the habitation of patients in their new country.

## Conclusion

The challenges of providing good quality care to patients are immense. As health care systems continue to develop, they become more complex [31]. They introduce new systems, rules, regulations and technology such as AI, [32] all while experiencing restrictions on resources such as space, staffing, and finances. This presents a challenge to the underlying purpose of HCPs and their sites of operation [33]. This challenge is brought into relief when one views the medical context through the lens of philosophers such as Levinas and Derrida. Here the hospital reclaims its linguistic roots to provide hospitality while HCPs take on the role of emplaced hosts who have constructed for themselves a dwelling in a foreign land. Patients are recast, not as performance metrics [34, 35] to be used to measure the success or failure of a HCP or site of care, but as sojourners: strangers to the land of illness and disease. Patients find themselves cast adrift in an alien world that threatens their inner stability and security. Consequently they seek out oases; places of refuge, an opportunity to recollect themselves, and the potential to build their own shelter.

Viewing medical care contexts through the lens of Levina’s absolute priority of the Other and Derrida’s law of unconditional hospitality challenges HCPs to open up their dwellings in an act of self-sacrifice. As HCPs respond with hospitality to patients they break their pagan shrines of self-isolation, viewing their dwellings, not as bunkers to be rooted into, but as places from which they may reach for the object of their metaphysical desire: the transcendent Other. Such dwellings come in many forms, but arguably one of the most important is that of the non-physical mobile home of language.

Paradoxically, this mobile site may also pose a threat to vulnerable patients. Just as hospitality contains within it the roots of its anti-thesis hostility, language presents to the patient a door that emphasises not only its openness, but its exclusionary potential. Those who control the mobile site of language have the keys to the door, they may use this door to invite patients in, teach them how to use appropriate language for their journey through the land of illness and disease (medical jargon), and help patients to build their own non-physical mobile dwellings. On the other hand, the masters of this site may laude it over guests, use their mastery to subsume patients under their own categories, use patients for their own purposes – as a means to a selfish end. Doing so will only further enroot HCP in their own self-constructed pagan shrine. More than this, it will emphasise to patients the strangeness of the land of illness and disease, and the hopelessness of finding a pathway through it.

This understanding of the medical context emphasises both the possibilities and dangers of opening our sites of care to patients. It reminds HCP of the universal searching for hospitality that we all experience, but in particular, patients who find themselves vulnerable. Yet this construction encourages more. It encourages HCP to take up Derrida’s call to do the impossible in so far as it is possible [4]: to offer a door to the Other in such a way that they can cross the threshold without fear. To allow the other to enter with the confidence of finding, not only a place of refuge for their immediate suffering, but the context to learn to use the linguistics tools necessary to build their own mobile home in the land of illness.

## Data Availability

NA.

## Footnotes

1 Authors work redacted for anonymity.
